# Sex and age predict habitat selection in the world’s most geographically extensive lion population

**DOI:** 10.1007/s00442-025-05744-x

**Published:** 2025-07-02

**Authors:** Dominik T. Bauer, Genevieve E. Finerty, M. Kristina Kesch, Christos Astaras, Robert A. Montgomery, David Heit, Joerg U. Ganzhorn, David W. Macdonald, Andrew J. Loveridge

**Affiliations:** 1 Wildlife Conservation Research Unit, Department of Zoology, University of Oxford, Tubney House, Abingdon Road, Recanati-Kaplan CentreTubney, Oxfordshire OX13 5QL UK; 2 Forest Research Institute, Hellenic Agricultural Organization “Demeter”, TK57006 Thessaloniki, Vasilika Greece; 3 Research On the Ecology of Carnivores and Their Prey Laboratory, Department of Fisheries and Wildlife, Michigan State University, East Lansing, Michigan 48823 United States of America; 4 Department of Animal Ecology and Conservation, Hamburg University, Martin‐Luther‐King Platz 3, 20146 Hamburg, Germany

**Keywords:** *Panthera leo*, Resource selection, Landscape conservation, Demographic differences, KAZA TFCA

## Abstract

Conservation of large carnivore populations requires effective management strategies that promote landscape-scale protection and genetic connectivity. Pivotal to the success of these strategies is sufficient evidence, including quantifying the processes that govern species distribution. We used telemetry data from 63 lions from the Kavango-Zambezi Transfrontier Conservation Area (KAZA TFCA) in southern Africa to analyze inter-demographic differences in habitat relationships using a mixed-effects resource selection analysis approach. In this semi-arid landscape, some of the most important drivers of habitat selection are surface water and precipitation, which in turn regulate prey abundance. Predicted relative probability of habitat selection was highest near water irrespective of age and sex; however, the effect of precipitation varied depending on the demographic class. Adult lions and subadult females preferred habitat with above average rainfall; however, the opposite was true for subadult males which showed a strong aversion to precipitation. Across all four demographic classes, relative probability of habitat selection was generally positively associated with higher levels of prey abundance with the exception of gemsbok which was negatively correlated with adult female, subadult male, and subadult female habitat use. The predicted distributions for all four demographic classes were widespread across multiple different land-use types, highlighting the need to extend the traditional concept of formally protected areas to include multi-use landscapes and support large-scale transboundary conservation initiatives.

**Supplementary Information:**

The online version contains supplementary material available at 10.1007/s00442-025-05744-x.

## Introduction

In the face of rapidly increasing anthropogenic pressure, overexploitation of natural resources, habitat loss, and fragmentation, driven by the expansion of agriculture and human settlements, are among the main drivers of biodiversity decline (Maxwell et al. 2016; Jaureguiberry et al. 2022). Given the persistence of these threats, it is essential to understand and predict how animals respond and adapt to changes in their environment to mitigate population declines. Wide-ranging organisms such as large carnivores are particularly affected, and are forced to compete with humans for suitable habitat (Valenzuela-Galván et al. 2008), often resulting in human–wildlife conflict and population decline (Ripple et al. 2014). Persistence of large carnivore populations requires effective management and protection strategies that promote landscape-scale protection and genetic connectivity (Björklund 2003; Balbuena-Serrano et al. 2021). Pivotal to the success of these strategies is sufficient evidence, including the quantification of the processes that govern species distributions (Sutherland et al. 2004).

Habitat selection in large carnivores is influenced by several bottom–up and top–down factors. Carnivore densities generally reflect prey abundance in natural ecosystems (Carbone and Gittleman 2002; Hayward et al. 2007), which in turn is regulated by primary productivity (Hopcraft et al. 2010) and water distribution and availability (Valeix et al. 2010). While some members of the order *Carnivora* can coexist successfully and even thrive alongside humans (Kuijper et al. 2016), others such as the African lion *(Panthera leo)* or African wild dog *(Lycaon pictus)* are sensitive to anthropogenic pressures (Abade et al. 2020; O’Neill et al. 2020). These can include killings in response to human–wildlife conflict (Kissui 2008) due to cultural norms and beliefs (Dickman et al. 2014; Carter et al. 2017) as well as non-lethal activities such as unsustainable bushmeat harvesting that depletes natural prey populations (Everatt et al. 2014) and incompatible land use that can alter behavior, distribution, and survival rates (Dheer et al. 2022).

Resource selection functions (RSFs) are a popular tool to identify critical resources for animal populations by comparing habitat characteristics at locations animals use to those that are theoretically available, or unused, by the animal (Manly et al. 2007; Millspaugh et al. 2014). When applying RSFs, it is beneficial to account for sex and age, as different demographic classes within a species often have distinct ecological needs (Elliot et al. 2014; Marchand et al. 2014; Rossman et al. 2015). This approach can inform relevant conservation actions and has been successfully applied in various contexts, such as managing coastal development to protect sea turtles (Tucker 2010), implementing age-specific habitat restoration projects for Atlantic salmon *(Oncorhynchus nerka)* population recovery (Hinch et al. 2012), and devising human–wildlife conflict mitigation measures for male lions (Patterson et al. 2004).

We used a mixed-effects RSF analysis approach to account for differences between sex and age class on a large telemetry dataset of the African lion from the Kavango-Zambezi Transfrontier Conservation Area (KAZA TFCA), a mixed-use landscape in southern Africa covering parts of Angola, Botswana, Namibia, Zambia, and Zimbabwe. The aim of our study was to assess how a suite of environmental and anthropogenic landscape variables, as well as relative prey abundance, influence space use of four demographic classes of lions (adult female, subadult female, and adult male and subadult female) in this multi-use landscape.

Female lions are largely philopatric with young females generally staying in their natal pride, only dispersing when potential pride size exceeds the habitat-specific optimum (Packer and Pusey 1987; VanderWaal et al. 2009). Adult male lions, which often have to defend territories and compete with other males for pride control, show distinct habitat preferences that support their territorial and reproductive strategies. Their habitat use is influenced by the need to patrol large territories and access to females and prey. In contrast to females and adult males that exhibit territorial and residential behavior, subadult males are not mature enough to challenge for a pride, and exhibit non-territorial behavior (Elliot et al. 2014), relying on areas of marginal habitat quality that might be less suitable but offer refuge from adult males. Consequently, we expected differences in habitat selection between sexes and most notably subadult males due to their different resource needs compared to the other demographic classes.

We hypothesized that lion occurrence, irrespective of demographic class, would be positively influenced by the proximity to water sources due to the semi-arid nature of the landscape. We also expected habitat productivity to positively influence lion habitat selection across all demographic classes, with variations in preference intensity, reflecting each class's specific ecological needs. Relative prey abundance was anticipated to positively correlate with habitat selection for all demographic classes; however, we expected the strength and specific prey preferences to vary. We hypothesized that anthropogenic factors such as proximity to human settlements to negatively influence habitat selection due to the risk of human–wildlife conflict. We expected the degree of this influence to differ among demographic groups, with subadult males expected to show higher tolerance for marginal habitats near human activity zones. To test this, we use a mixed-effects RSF analysis modeling approach to predict relative probability of habitat selection for each demographic class across the study area. We discuss the implications of our findings for the management and protection of this geographically critical habitat and highlight its importance to the ecological functionality of the KAZA TFCA.

## Materials and methods

### Study area

**Fig. 1:**
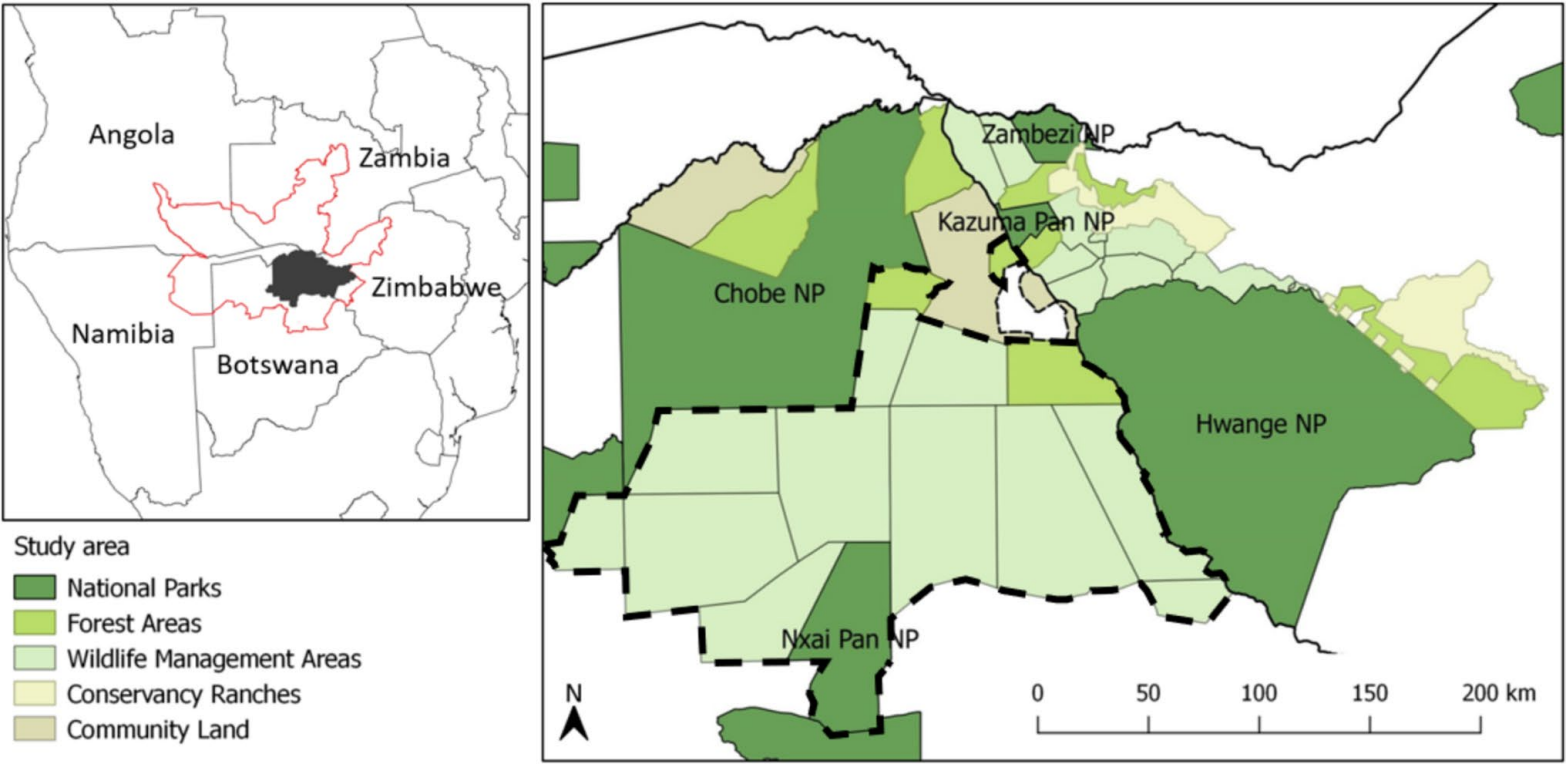
Study area with land-uses inset, its extent (in dark gray) within the KAZA TFCA (red outline) (top left). The dashed line shows the extent of the area where track counts were conducted

We situated our study in a ~ 70,000 km^2^ section of the KAZA TFCA at the border of Botswana and Zimbabwe (between 17°47’ – 20°15’S and 23°32’ – 27°44’E; Fig. 1). There are five national parks, 10 forest reserves, 19 wildlife management areas, as well as 20 conservancy ranches, communal areas, and high-intensity farming blocks in our study area. The area experiences two distinct seasons; a dry season from April to October, and a rainy season from November to March. Annual mean precipitation ranges from 680 mm in the northeast to around 400 mm in the southwest (Batisani and Yarnal 2010; Mazvimavi 2010). In this semi-arid landscape, there is no natural surface water during the dry season other than the perennial Chobe and Zambezi Rivers to the north and the Okavango Delta to the west of the study area. However, water is available at over 250 artificial waterholes, of which more than 80% are located in Zimbabwe. Tree and shrub savannah vegetation is dominated by *Baikiaea plurijuga* in the north, *Combretum spp*., *Acacia spp*., and *Terminalia sericea* in the south, and *Colophospermum mopane* in the southwest.

### Covariates

#### Environmental and anthropogenic

Based on a priori rationale, we considered seven site-specific covariates to affect lion distribution across the study area (Table 1). Specifically, we considered covariates associated with primary productivity, including mean annual precipitation *(Precipitation)*, percent canopy cover *(Canopy),* and Normalized Difference Vegetation Index *(NDVI)*. We accounted for water availability by calculating distance to the nearest waterhole or perennial river *(Distance to Water)* as well as human disturbance by calculating distance to the nearest settlement *(Settlement Distance).* To correctly represent the impact of population density on the surrounding environment, we created point vectors of houses (mean household size 3.7) (Statistics Botswana 2012) and used the QGIS v.2.14 (QGIS Development Team 2016) kernel density estimation algorithm (within the heatmap plugin), with a 10,000 m radius and a quartic (biweight) kernel *(Settlement Density).* The same process was repeated for waterholes and rivers which were treated as a series of waterholes 100 m apart *(Water Density).* We developed a database of these different geospatial layers representing each covariate as a raster using QGIS v.2.14 and online available (e.g., MODIS, percent canopy cover, NDVI), field collected (i.e., waterholes, roads), and manually digitized data (settlements).

**Table 1:** Covariates hypothesized to influence relative probability of habitat selection by lions across the study area

Covariate	Description	Resolution	Source
Environmental			
*Precipitation*	Mean annual precipitation (mm)	1000 m	http://worldclim.org
*Canopy*	Percent canopy cover	250 m	MODIS MOD44B http://glcf.umd.edu/data/vcf
*NDVI*	Normalized Difference Vegetation Index	250 m	MODIS MOD13Q1 https://modis.gsfc.nasa.gov
*Distance to water*	Distance to nearest available dry season surface water (m)	100 m	this study—Euclidean distance to the nearest waterhole (rivers treated as series of waterholes spaced 100 m apart)
*Water density*	Relative density of surface water per 100m^2^	100 m	this study—kernel density estimation algorithm with 10.000 m radius and a quartic (biweight) kernel using the heatmap plugin in QGIS v.2.14
Anthropogenic			
*Distance to settlement*	Distance to nearest human settlement as a proxy of anthropogenic impact (m)	100 m	this study—Euclidean distance to the nearest point vector of house
*Settlement density*	Relative density of houses per 100m^2^	100 m	this study—kernel density estimation algorithm with 10.000 m radius and a quartic (biweight) kernel using the heatmap plugin in QGIS v.2.14

### Prey distribution

To determine prey distribution across the study area, we employed vehicle-based track counts, with the assistance of highly qualified San (Bushmen) trackers (see Kesch et al. 2014). All trackers participating in the study had several years of tracking experience and were thoroughly trained for this particular type of data collection. Between Oct 2012 and Oct 2015, we recorded tracks of nine primary prey species of lions across the study area to be analyzed in an occupancy modeling framework (see Fig. 1). These species included African buffalo (*Syncerus caffer*), common eland *(Taurotragus oryx),* gemsbok *(Oryx gazella)*, giraffe *(Giraffa camelopardalis)*, impala (*Aepyceros melampus*), greater kudu *(Tragelaphus strepsiceros)*, warthog (*Phacochoerus africanus*), blue wildebeest (*Connochaetes taurinus*), and Burchell’s zebra (*Equus quagga*)*.* While the use of track-based indices to estimate species abundance has recently come under scrutiny (Dröge et al. 2020), in this study we merely recorded the presence and absence of species to model in an occupancy framework.

### Occupancy modeling

Occupancy modeling provides a method to account for imperfect detection (false absences) by repeatedly visiting survey sites (using detection histories that are created by using spatial or temporal replicates). We divided a posteriori the surveyed road network with a total transect length of 4,588.97 km into 201 road segments (mean length 22.8 km (SE = 0.87) using an 8*8 km grid across the entire landscape. To create the detection histories for occupancy modeling, we split the road sections within each grid cell into 2 km segments and treated each of them as a spatial replicate where the presence or absence of the respective species was recorded. To avoid spatial dependence of detections in consecutive road segments (sites), we stepwise increased the segment length by 2 km until the single-season occupancy model without Markovian dependence (MacKenzie et al. 2002) proved a better fit, outperforming the occupancy model with Markovian dependence (Hines et al. 2010) by at least two Akaike Information Criterion (AICc) values (Anderson and Burnham 2004). Since there was considerable difference in track encounter rate across sampling units, we assumed that the detection probability was also influenced by variation in animal abundance. We therefore opted to apply an abundance-induced heterogeneous detection probability model (Royle and Nichols 2003).

Again, based on a priori rationale, we considered nine site-specific covariates in two categories (Environmental and Anthropogenic) that could affect prey distribution across the study area (Table S1, Supporting Information). Covariates in the Environmental category corresponded to landscape productivity (e.g., carbon and nitrogen soil content, vegetation cover, water availability, and precipitation), while covariates in the Anthropogenic category corresponded to human disturbance (distance to nearest settlement and settlement density). Using the R package “unmarked” (Fiske and Chandler 2011) in R version 3.5.2 (R Core Team 2018), we fitted single-season, single-species occupancy models with abundance-induced heterogeneous detection probability to our detection histories. We began the modeling process by first considering covariates that would influence the detection probability of an individual (r) while keeping the mean expected abundance (λ) constant. Since substrate quality across the study area was homogenous and roads were rarely if ever driven there was no need to test any other detection covariates apart from the number of 2 km segments needed to achieve spatial independence. Candidate models were built by testing each covariate from the Environmental and Anthropogenic category using univariate analysis retaining only covariates that had a significant effect on occupancy. We tested for correlation between the remaining covariates and eliminated the one with higher AICc when collinearity was observed (|r|> 0.70). We then ran a multivariate model that included all possible combinations of non-collinear covariates identified as important in the univariate analyses and assessed the goodness of fit using Pearson’s chi-square statistic with 1000 parametric bootstraps in the R package AICcmodavg (Mazerolle 2019). Models with ΔAICc ≤ 2 received equal support and we used the MuMIn package in R (Barton 2015) for conditional model averaging. We used the top models’ covariate coefficients to estimate the relative probability of prey intensity of habitat use across the study area.

### Resident lion data

#### GPS collar data

Between 2012 and 2016, we fitted GPS telemetry collars to a total of 63 lions (14 adult males, 20 adult females, 22 subadult males, and 7 subadult females). In the post-processing of the telemetry data, we filtered for impossible movements and used a biologically informed threshold to exclude non-residential movement. See supporting information for a detailed description of the process.

### Resource selection functions

Emulating common input variables available to field biologists in assessing habitat selection, we created two candidate sets of variables for our RSF models: *environmental* and *anthropogenic* variables (from remote sensing; see Table 1), and *prey* (from occupancy models) only. We ran two RSFs (one for each candidate set) with a used-available design, hereafter referred to as *environmental model* and *prey model*, respectively. We completed all analysis and data processing for the RSFs in R.

For each individual lion, we calculated utilization distributions (UDs) using kernel density estimators to create a spatial extent for third-order resource selection analysis. We used a bivariate plugin matrix (Gitzen et al. 2006) calculating separate bandwidths to generate contiguous 95% annual dry season home ranges (Apr–Oct) for each lion with at least 2 months of positional data. We then used estimates of date of birth (estimated based on physical examination of each animal during the collaring process or from project records of known age lions) to assign each seasonal home range to one of four demographic classes: adult female, subadult female, adult male and subadult male (subadult ≤ 4 years), based on age at the start of the season. We considered observed locations (*n* = 40,271) as “used” and randomly generated available locations within 95% UDs while maintaining a 1:4 ratio of “used” vs “available” (Millspaugh et al. 2014). We extracted values of the candidate explanatory variables under both “used” and “available” points using the raster package in R to generate our datasets for the RSF analyses.

For each RSF, we fit a logistic generalized linear mixed model using the R package “lme4” (Bates et al. 2014). We fit a random intercept for home range within individuals and random slopes for each covariate for home range within individuals to account for repeated sampling of individuals, and from seasonal home range within individuals. We considered the inclusion of random intercepts, random slopes, and the correlation between these variance components. To optimize our random-effects structure, we followed the procedure from Bates et al. (2018), where a global model (in terms of random-effects structure) balances the inclusion of adequate components with the fitting of over-specified random effects given the information in the data.

As our goal was to understand the relative effect of individual variables on lion habitat selection (rather than a predictive model), we took steps to avoid collinearity between our predictor variables to facilitate simple interpretation of model coefficients. We standardized and centered all input variables. For each both RSF models, we tested for correlation between the candidate explanatory variables and stepwise eliminated the covariates with the highest Variance Inflation Factor values until the remaining ones dropped below the critical threshold of three (Zuur et al. 2010). We then built our maximal models to include these remaining variables as fixed effects, alongside a two-way interaction with our age-and-sex category for each variable. We assessed the statistical significance of model terms (at the 0.05 level) via single term deletions from the full model using likelihood ratio tests. We assessed higher order terms (interactions between variables) first, followed by main effects after the removal of any non-significant interaction terms. A significant interaction term in this context would indicate differential habitat selection between our demographic classes for that variable.

Our environmental model allows us to explore the impact of different broad-scale habitat variables, which are predicted to be important for lions either as hot spots of productivity (across prey species) or as sources of risk and/or disturbance. The prey models allow us to explore in more detail the impact of areas of habitat with high predicted relative abundance of the individual key prey species for lions. While comparing between models is not the main focus of the study, we provide AIC and R^2^ statistics for the final models as context on relative model performance.

## Results

On average, we tracked adult females for 1.44 dry seasons (range 1–3), subadult females for 1.66 (range 1–3), adult males for 1.42 (range 1–4), and subadult males for one dry season. After post-processing of the telemetry data, we retained 3,875 daily locations for adult females, 1,859 for subadult females, 3,261 for adult males, and 1,789 for subadult males. We used these to develop 52 UD estimates for 36 individual home ranges.

Track count transects covered 474 sites with an average of 9.68 km per site, which resulted in a total transect length of 4,598 km. For each species, spatial independence of detections in consecutive road segments was achieved at 6 km. None of the final models indicated lack of fit (*p* value > 0.05) or showed signs of overdispersion (ĉ < 1.7). Maps predicting the spatially explicit relative abundance using the top model beta coefficients for each species can be found in the supporting information (Fig. S2, Supporting Information).

### RSFs

#### Environmental model

All candidate explanatory variables in the final environmental model were significant predictors for relative probability of habitat selection (Table 2). For *Distance to Water*, *Distance to Settlement,* and *NDVI,* the data were best described by a single slope across all demographic classes; however, selection in response to *Precipitation* was described best with separate slopes for each demographic class, as shown by the significant interaction term.

**Table 2:** In environmental model, fixed effects output from generalized mixed-effects model describing the predicted relative probability of habitat selection as a function of environmental and anthropogenic covariates and demographic category. Estimated effect sizes and their standard errors are presented, with significance of removal of term assessed using likelihood ratio tests. All continuous covariates have been scaled and centered, indicated by a z. prefix before covariate name. All coefficients are relative to the reference category “adult females”

	Wald Z test				Likelihood ratio test			
	β	SE	Z-statistic	P_z_	OR (95% CI)	df	χ^2^	Pr(χ^2^)
(Intercept)	− 1.56	0.26	− 5.93	< 0.001				
subadult females	− 0.37	0.47	− 0.78	0.44				
adult males	− 0.85	0.42	− 2.00	0.05				
subadult males	0.35	0.46	0.75	0.45				
z.*Distance to Water*	− 0.98	0.12	− 8.20	< 0.001	0.37 (0.29–0.47)	1	38.88	< 0.001
z.*Distance to Settlement*	− 0.25	0.10	− 2.44	0.01	0.77 (0.64–0.94)	1	5.45	0.020
z.*NDVI*	− 0.74	0.12	− 6.05	< 0.001	0.47 (0.37–0.60)	1	24.74	< 0.001
z.*Precipitation*	0.59	0.31	1.88	0.06	1.80 (0.98–3.27)	3	9.98	0.019
z.*Precipitation*:subadult females	− 0.18	0.30	− 0.60	0.55	0.83 (0.46–1.50)			
z. *Precipitation*:adult males	0.19	0.49	0.39	0.70	1.21 (0.45–3.10)			
z. *Precipitation*:subadult males	− 1.30	0.54	− 2.39	0.02	0.27 (0.09–0.78)			

^*^ Model statistics: AIC 36878.3 I Marginal R^2^ 0.25 I Conditional R^2^ 0.63

**Fig. 2:**
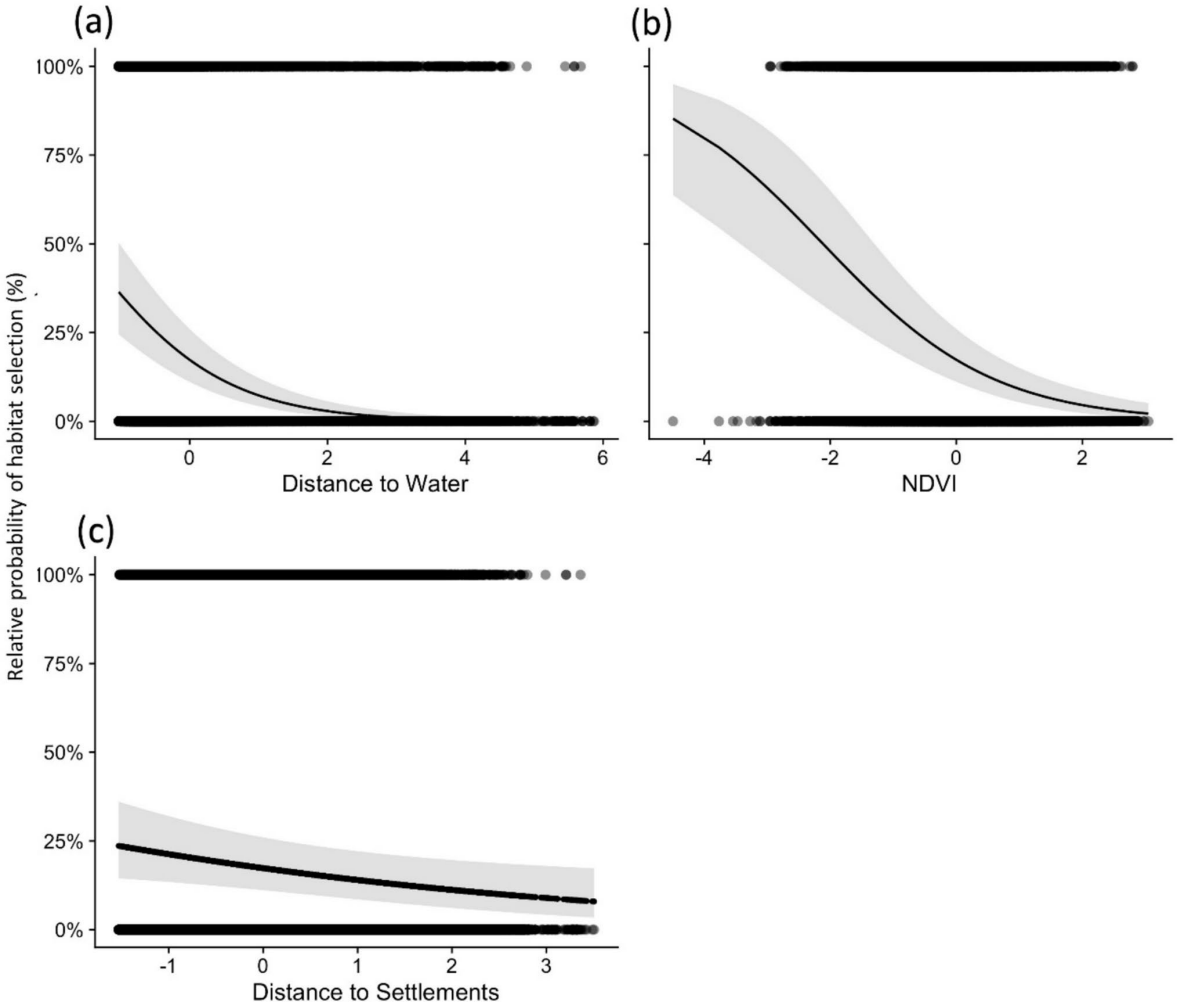
Relative probability of habitat selection by lions (with 95% CI) in relation to variation in **a** distance to water, **b** NDVI, and **c** distance to settlements. Note that all variables are standardized with a mean of zero and a standard deviation of one

**Fig. 3:**
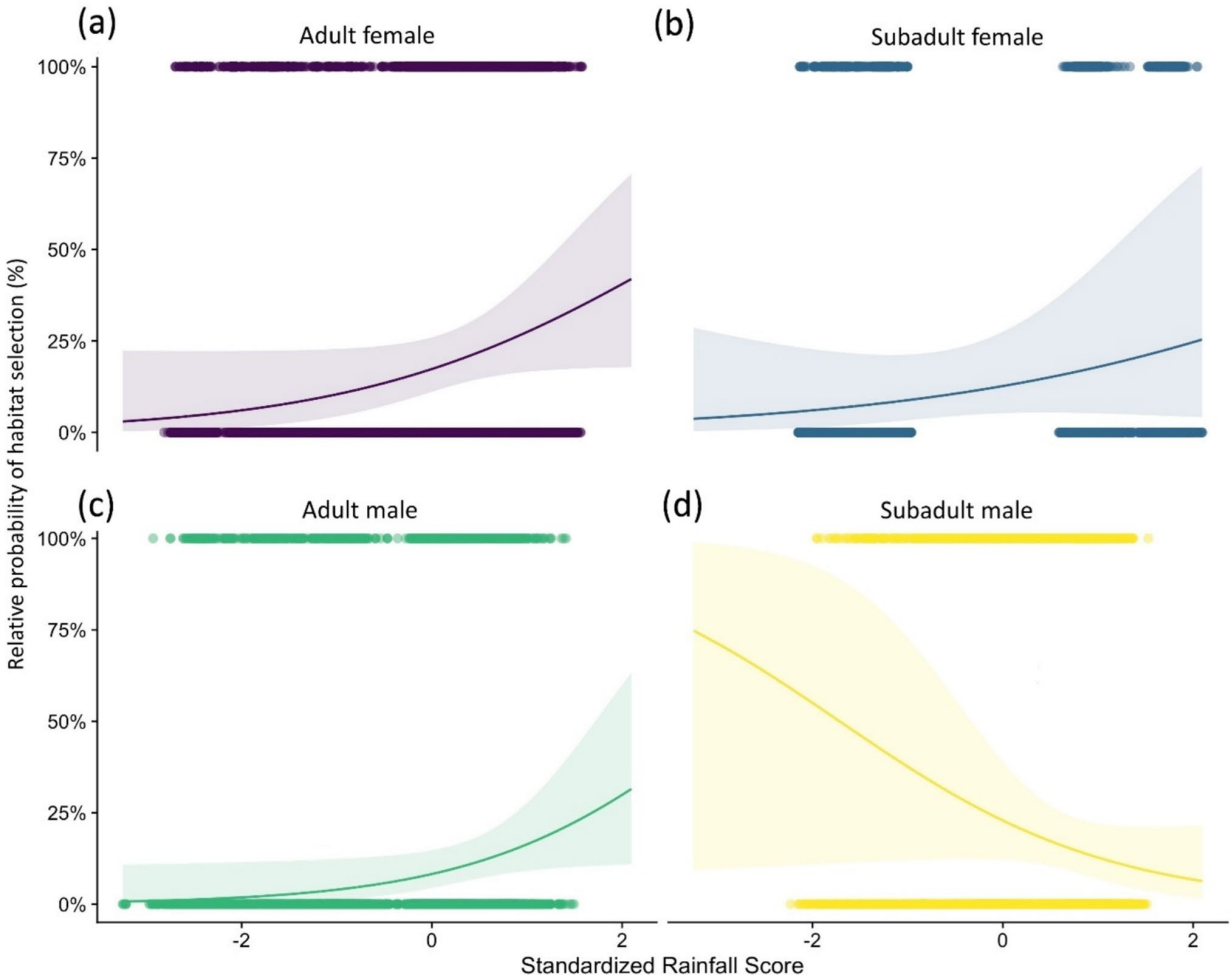
Relative probability of habitat selection by lions (with 95% CI) for **a** adult females, **b** subadult females, **c** adult males, and **d** subadult males given variation in mean annual precipitation. Note variable is standardized with a mean of zero and a standard deviation of one

**Fig. 4:**
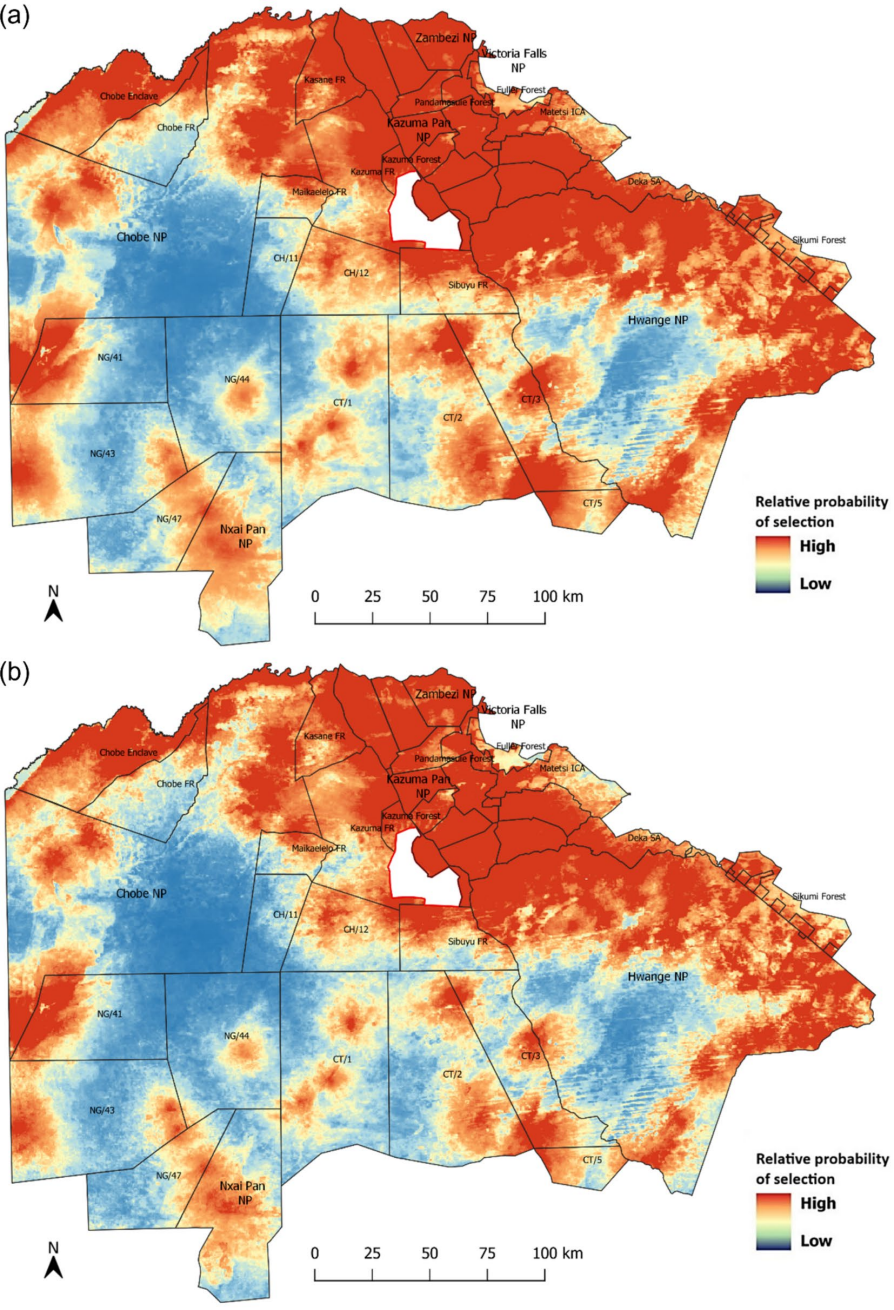

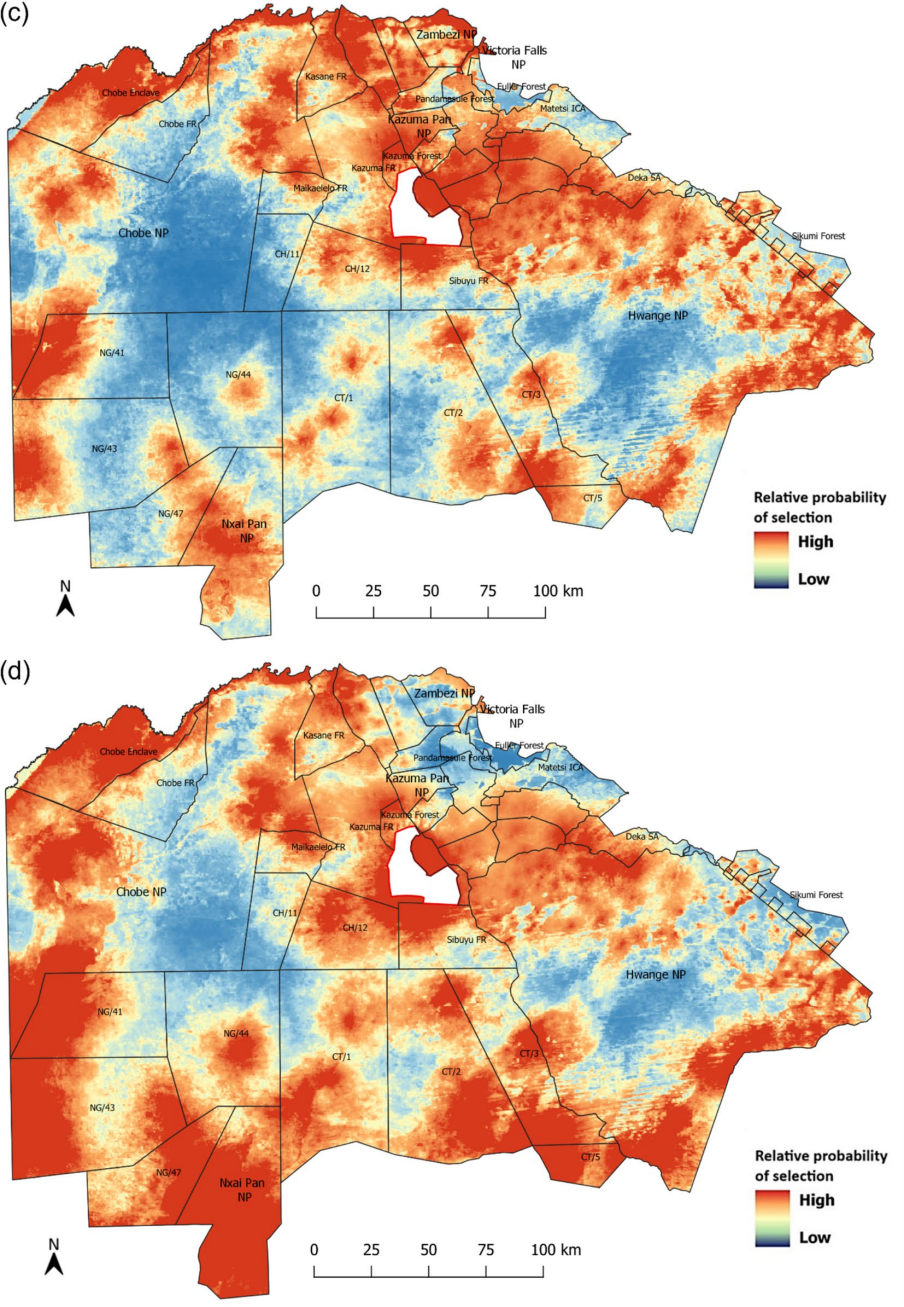
Relative probability of habitat selection by lions for **a** adult females, **b** subadult females, **c** adult males, and **d** subadult males using the model-averaged coefficients from the environmental model

Predicted relative probability of habitat selection was reduced with increased distance to surface water (Fig. 2a) and significantly higher in open grassland (low *NDVI*) than dense forest (high *NDVI*) (Fig. 2b). Furthermore, the model suggested that habitat selection decreased slightly with increasing distance from settlements (Fig. 2c). Whereas the model suggested that precipitation had a positive impact on habitat selection of adult females, subadult females, and adult males, the relationship was opposite for subadult males which seem to not select areas with higher rainfall (Fig. 3). We developed maps depicting the relative probability of habitat selection for each demographic class (Fig. 4) using the model-averaged coefficients from the environmental model.

### Prey model

**Fig. 5:**
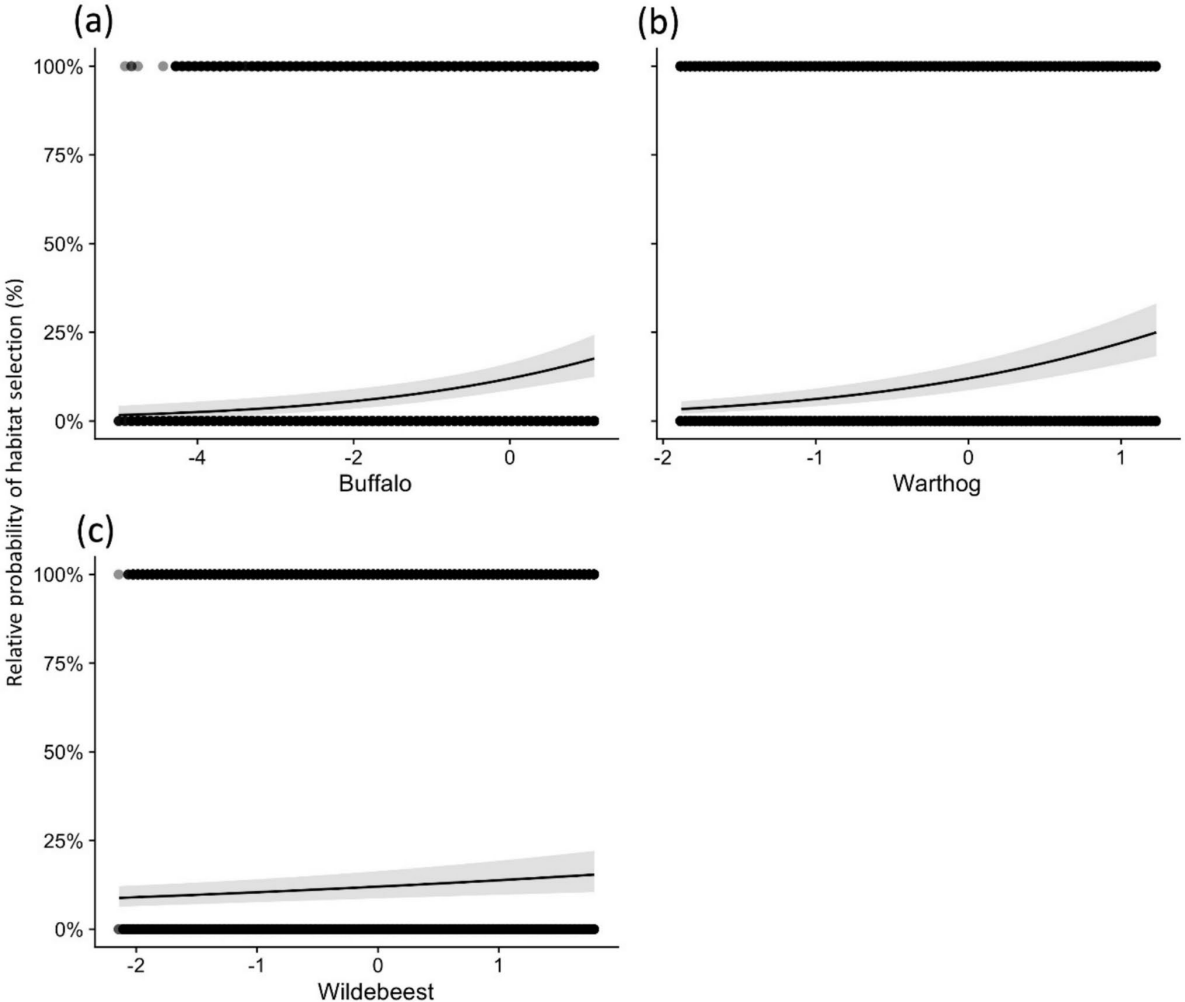
Relative probability of habitat selection by lions (with 95% CI) given variation in **a** buffalo, **b** warthog, and **c** wildebeest densities. Note that all variables are standardized with a mean of zero and a standard deviation of one

**Fig. 6:**
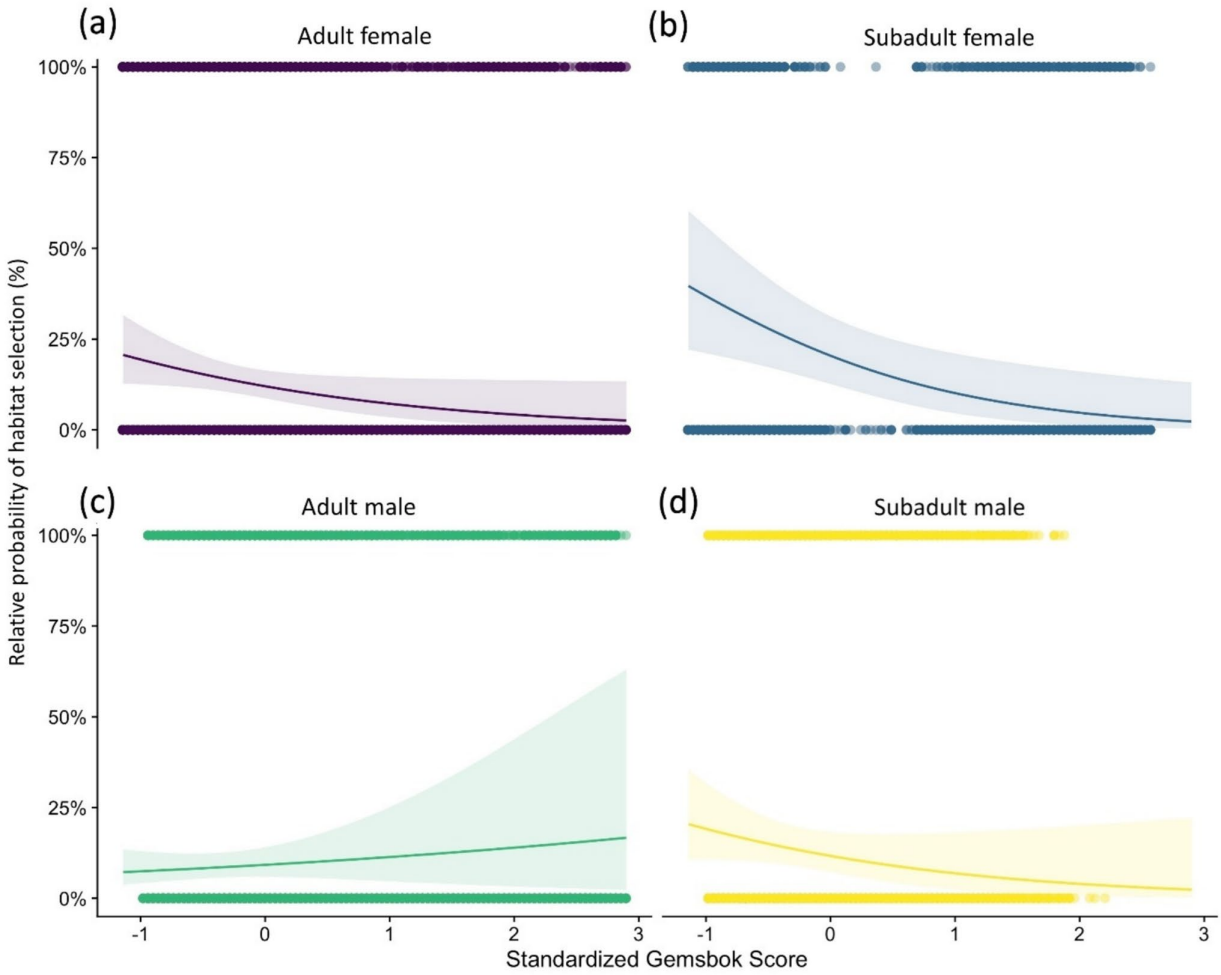
Relative probability of habitat selection by lions (with 95% CI) for **a** adult females, **b** subadult females, **c** adult males, and **d** subadult males given variation in gemsbok densities. Note variable is standardized with a mean of zero and a standard deviation of one

All covariates in the final prey model had a significant effect on the predicted relative probability of habitat selection by all four demographic classes (Table 3). The top model included *Eland* and *Gemsbok* with a group-level interaction with separate slopes for each demographic class, and *Buffalo*, *Warthog*, and *Wildebees*t without a random effect. The model revealed that predicted relative probability of habitat selection correlated positively with increasing relative abundance of *Buffalo* (Fig. 5a), *Warthog* (Fig. 5b), and *Wildebeest* (Fig. 5c). Relative *Eland* abundance seemed to have a relatively weak effect on habitat selection despite some variation between demographic classes. The overall effect of relative *Gemsbok* abundance on relative probability of habitat selection by lions was strong but varied considerably between classes, with adult males seeming to prefer habitat with relatively higher *Gemsbok* numbers (Fig. 6).

**Table 3:** In Prey model, fixed effects output from generalized mixed-effects model describing the predicted relative probability of habitat selection as a function of prey covariates and demographic category. Output is displayed as in Table 1

			Wald Z test			Likelihood ratio test		
	β	SE	Z-statistic	P_z_	OR	df	χ^2^	Pr (χ^2^)
(Intercept)	− 1.99	0.18	− 10.83	0.00				
subadult females	0.63	0.29	2.16	0.03				
adult males	− 0.30	0.29	− 1.02	0.31				
subadult males	− 0.03	0.32	− 0.10	0.92				
z.*Buffalo*	0.42	0.09	4.55	0.00	1.52 (1.27–1.81)	1	14.38	< 0.001
z.*Eland*	0.05	0.10	0.51	0.61	1.05 (0.86–1.27)	3	10.28	0.016
z.*Eland*:subadult females	− 0.29	0.12	− 2.30	0.02	0.74 (0.59–0.94)			
z.*Eland*:adult males	− 0.40	0.16	− 2.47	0.01	0.67 (0.49–0.91)			
z.*Eland*:subadult males	− 0.03	0.18	− 0.15	0.88	0.97 (0.68–1.38)			
z.*Gemsbok*	− 0.57	0.28	− 2.03	0.04	0.56 (0.32–0.98)	3	8.90	0.031
z.*Gemsbok*:subadult females	− 0.26	0.15	− 1.72	0.09	0.77 (0.57–1.03)			
z.*Gemsbok*:adult males	0.80	0.38	2.12	0.03	2.22 (1.05–4.66)			
z.*Gemsbok*:subadult males	− 0.02	0.40	− 0.04	0.97	0.98 (0.44–2.10)			
z.*Warthog*	0.73	0.09	8.03	0.00	2.07 (1.73–2.46)	1	34.40	< 0.001
z.*Wildebeest*	0.16	0.05	3.31	0.00	1.17 (1.06–1.30)	1	7.65	0.006

^*^ Model statistics: AIC 37242 **I** Marginal R^2^ 0.17 **I** Conditional R^2^ 0.51

## Discussion

With growing anthropogenic pressure and habitat loss threatening natural ecosystems, it is essential to understand and predict how animals respond to variation in their environment. RSFs are a useful tool for evaluating these responses and provide valuable insights into the biotic and anthropogenic factors that determine the distribution of carnivores across an extensive landscape. We show that in this semi-arid landscape, surface water and precipitation which in turn regulate prey abundance are some of the most important drivers of habitat use for lions. We highlight the differences in response between demographic classes and thereby contribute to a growing body of knowledge on large carnivore species ecology which is essential for their effective conservation and management.

### Environmental and anthropogenic drivers

Our results show that habitat use of lions was highest in close proximity to water irrespective of age and sex. These findings are supported by the previous studies that show that water availability strongly influences the distribution, density, and behavior of animals in a semi-arid environment (Rozen-Rechels et al. 2015). In landscapes with limited access to surface water, many herbivore species must meet their nutritional requirements within the constraints set by the location of water sources, which predators use to their advantage (Rosas-Rosas et al. 2008). While lions are relatively independent of water (Eloff 1973b), studies have shown that they actively select areas in proximity to waterholes (Valeix et al. 2010) which play a key role in shaping their home ranges.

Apart from the perennial Chobe and Zambezi Rivers to the north and the Okavango Delta to the west of the study area, all available water is provided through artificial water sources. The provision of artificial water is a common management tool and has been widely applied in southern Africa to increase the abundance and distribution of ungulates, and mitigate the impact of man-made barriers blocking access to dry season water sources (Selebatso et al. 2018). While the provision of artificial water has been shown to expand the distribution of common water-dependent species, such as buffalo, zebra, and blue wildebeest (McLoughlin and Owen-Smith 2003), the negative effects on biodiversity have been demonstrated in multiple studies (Owen-Smith 1996; Parker and Witkowski 1999; Grant et al. 2002). Under natural conditions, large herbivores need to move frequently between locations that offer sufficient water and forage. The provision of water encourages herbivores to become sedentary (Mills and Retief 1984), which generates pressure on the surrounding habitat and leads to range degradation (Thrash 1998).

The effects of water provision on the herbivore and plant communities have been well documented in recent years (Western 1975; Thrash 1998; Chamaillé‐Jammes et al. 2007); however, its influence on predator populations is less well understood. The increase in herbivore populations likely boost predator numbers in the short and medium term (Smuts 1976; McLoughlin and Owen-Smith 2003), but the impact of habitat changes and its implications for the long-term sustainability of predator–prey systems is unclear.

The year-round provision of water in Hwange National Park (an area with little natural surface water) has certainly contributed to its attractiveness as a tourism destination, with the income generated supporting the long-term protection of its flora and fauna. Furthermore, the artificial waterholes in the wildlife management areas of Botswana have allowed water-dependent prey species and therefore lions to access areas previously too dry to support them, creating a large, connected population across the entire central KAZA TFCA region. Strategically placed waterholes could serve as a tool to promote landscape connectivity by strengthening (or even re-establishing) lion corridors. Maintaining a prescribed minimum distance between water sources or the rotational opening and closing of waterholes such as in Kruger National Park (Wyk 2001) would facilitate herbivore movement while allowing the vegetation to recover, thus avoiding habitat degradation. However, care has to be taken when placing waterholes in the vicinity of human settlements or near to the boundary of protected areas, as this can aggravate human–wildlife conflict and increase anthropogenic edge effects (Woodroffe and Ginsberg 1998).

As hypothesized, our results suggested that precipitation played an important role in the predicted relative probability of habitat selection by lions. The way in which lions responded to rainfall depended on an interaction with the demographic class. The effect of rainfall on net primary productivity and its positive correlation with total large herbivore biomass in arid savannahs (Coe et al. 1976) has been documented previously (Owen-Smith and Ogutu 2003), while carnivore densities correlate with the biomass of the preferred prey species or size class of prey (East 1984; Hayward et al. 2007). A study in the Serengeti showed that rainfall positively affected short-term reproductive success of lions (Mosser et al. 2009) and that population dynamics followed the variation in rainfall patterns (Packer et al. 2005). This relationship is reflected in our results which show that adult females, subadult females, and adult males preferred habitat with higher-than-average rainfall. For subadult males, the relationship was inverse, with the relative probability of habitat selection showing a strong negative correlation with annual precipitation. This supports our hypothesis and is most likely due to the fact that all of the subadult males collared in this study were between 28 and 48 months old—a time in which they approach or have reached sexual maturity and are pushed into marginal, less-productive areas by the stronger, territorial adult males (Loveridge et al. 2017).

With climate change expected to affect southern Africa particularly disproportionately, understanding how lions will use the landscape under changing climatic conditions is essential for their successful management. Projections indicate increased temperatures and more variable precipitation patterns, including both droughts and intense rainfall events (IPCC Climate Change 2019). These changes can have profound impacts on the ecosystems within this region, altering the availability of water and affecting primary productivity and prey abundance (Midgley and Bond 2015). Consequently, it is crucial to account for climate change and shifting rainfall patterns in conservation planning. Conservation strategies need to be adaptive, promoting multi-use landscapes and connecting areas of higher and lower rainfall to facilitate animal movement and resilience against climatic shifts. Enhancing landscape connectivity through transboundary conservation areas like the KAZA TFCA can help mitigate the impacts of climate change by providing corridors for wildlife to migrate between diverse habitats, thereby maintaining ecological processes and genetic diversity. Such integrative approaches are critical in a time when connectivity and protected area networks are heavily discussed as key components of effective wildlife conservation.

In recent years, NDVI has played a prominent role in ecological studies predicting animal distribution, movement, and life-history traits (Pettorelli et al. 2011; Abade et al. 2014). Our models revealed a strong negative correlation between NDVI and habitat use for lions. NDVI correlates with photosynthetically active plant biomass and vegetation productivity and is therefore commonly used as a proxy for the biomass of herbivores (Boone et al. 2006). With the distribution and densities of dominant carnivore species clearly linked to the biomass of suitable prey (Carbone and Gittleman 2002), their presence is commonly positively correlated with NDVI (Henschel et al. 2016).

In our study, the relative probability of habitat selection was highest in areas with low-to-moderate positive NDVI values (dry shrub and grassland) and lowest in areas with high NDVI values (woodland) for all demographic classes. The negative relationship may reflect the fact that the areas with the highest NDVI in the study area were teak forests, which are characterized by sandy and nutrient-poor soils and low forage quality in the dry season (Gambiza et al. 2008; Chidumayo and Gumbo 2010). Furthermore, the previous studies have recommended a careful approach when using NDVI as a proxy for vegetation in semi-arid and arid environments as it can be biased by soil reflectance (Asrar et al. 1984; Huete 1988), although others used it successfully even in sparsely vegetated areas (De La Maza et al. 2009).

In contrast to our hypothesis, our results indicated a positive correlation between habitat selection by lions and human settlements. While some predators have been shown to adapt to a more human-dominated environment (Fechter and Storch 2014; Braczkowski et al. 2018), lions are generally considered incompatible with most current livestock production practices (Rossell 2016) and avoid areas of human disturbance if possible (Mills et al. 2023). We believe that this result is most likely an artifact of the large number of artificial waterholes that have been placed in key tourism areas close to the border of Hwange National Park. With multiple human settlements on the adjoining communal farmland, this would explain why lions seemingly prefer areas with high human disturbance. This further highlights the need to exercise caution when establishing artificial waterholes on the edge of wildlife areas to avoid drawing predators into the vicinity of human settlements. Developing a cross-border policy framework to harmonize the management of man-made water sources across the TFCA would help balance the needs of its wildlife and communities.

### Prey

Although the environmental model had the strongest overall support based on AIC and marginal/conditional R^2^, the prey model remains ecologically informative. By including individual prey species from the occupancy survey, it enabled us to identify specific species–lion associations that are not detectable using broader proxies such as NDVI. Several prey covariates had among the largest standardized effect sizes of any variables in the analysis, highlighting the importance of prey availability in shaping lion space use in this landscape.

Our results confirm our hypothesis that the relative probability of habitat selection was generally positively associated with higher levels of relative prey abundance for all four demographic classes. This corroborates the previous findings that show that the distribution of dominant carnivore species is governed by the availability and biomass of suitable prey (Hayward et al. 2007; Davidson et al. 2019). Our findings are congruent with those of Davidson et al. (2013) that showed that buffalo and medium-sized Bovidae such as wildebeest were the most frequently utilized prey for male and female lions in the dry season. While warthog do not contribute such a significant proportion to their diet, the positive correlation between habitat selection of lions and warthogs’ relative abundance is most likely an artifact of warthogs dependence on water (D'Huart and Grubb 2001), which lions actively seek out for hunting grounds (Davidson et al. 2013).

Our results suggest that the relative abundance of eland is a significant predictor for the intensity of lion habitat use; however, compared to other predictors, the effect was relatively small. The number of eland track encountered was lower compared to the other species included in the models, and therefore, the final Royle–Nichols model did not show high variation. We believe that with a larger dataset, the relationship between relative abundance of eland and relative probability of habitat selection by lions might differ.

Relative abundance of gemsbok played an important role in the predicted relative probability of habitat selection by lions. As a water-independent species, gemsbok distribution was restricted to the southernmost part of the study area (see Figure S2, Supporting Information) which was completely devoid of surface water. Gemsbok have been shown to be a primary prey species for lions in the Kalahari ecosystem predominantly taken in the driest part of the ecosystem (Beukes et al. 2020). The positive correlation between the relative abundance of gemsbok and male habitat selection can most likely explained by the fact that adult gemsbok are a dangerous species to hunt (Eloff 1973a) with only the larger and most powerful lion males attempting to do so.

The significant role of prey availability in determining lion habitat selection underscores the necessity of maintaining robust prey populations for effective lion conservation. Overexploitation of prey species through bushmeat hunting and habitat degradation can lead to a decline in prey populations, subsequently impacting lion populations (Everatt et al. 2014). Therefore, ensuring sustainable prey populations through anti-poaching measures and habitat restoration is crucial. Integrated management strategies that include local communities can help in reducing illegal hunting and promoting sustainable land-use practices that benefit both wildlife and human livelihoods.

## Conclusion

The predicted distributions for all four demographic classes show the need to extend the traditional concept of formally protected areas to include multi-use landscapes and support initiatives such as TFCAs. Given that populations span across borders and boundaries, conservation area networks with sound land-use planning, emphasizing the needs of people and wildlife alike, are a promising movement to shape conservation in a human-dominated landscape. Enhancing landscape connectivity through corridors that link areas of high and low prey abundance can facilitate lion movement and access to essential resources, thereby promoting genetic diversity and enhance population resilience against climatic shifts. Our study area forms the core of the KAZA TFCA lion population (Cushman et al. 2018), and is part of what is evidently the geographically largest contiguous lion population left in the world, spanning across several hundred kilometers from Hwange National Park in Zimbabwe to the western parts of the Okavango Delta in Botswana. The insights we have gained on the biotic and anthropogenic factors influencing lion distribution at a landscape level provide an essential contribution to the development of an evidence-based strategy for lion conservation across the KAZA TFCA.

## Supplementary Information

Below is the link to the electronic supplementary material.

Supplementary file1 (DOCX 1273 KB)

## Data Availability

The datasets are available from the corresponding author.

## Code availability

The code is available from the corresponding author.
