# Supplementary material for: Sex and age predict habitat selection in the world’s most geographically extensive lion population
Source: Oecologia. 2025 Jul 2;207(7):120. doi: 10.1007/s00442-025-05744-x (PMC12222360; doi:10.1007/s00442-025-05744-x)
Supplement: Supplementary file 1 — Supplementary file1 (DOCX 1273 KB) [file 442_2025_5744_MOESM1_ESM.docx]

**Supporting Information**

Table S1 Spatially explicit covariates included in the occupancy modelling process for nine prey species

| **Covariate** | **Description** | **Resolution** | **Source** |
| --- | --- | --- | --- |
| **ENVIRONMENTAL** |  |  |  |
| ***Precipitation*** | Mean annual precipitation (mm) | 1000m | http://worldclim.org |
| ***VCF*** | Vegetation Continuous Fields  (percent canopy cover) | 250m | MODIS MOD44B  http://glcf.umd.edu/data/vcf |
| ***NDVI*** | Normalized Difference Vegetation Index | 250m | MODIS MOD13Q1  https://modis.gsfc.nasa.gov |
| ***Carbon*** | Organic Carbon  (g per kg at 5 cm depth) | 250m | Hengl et al., 2017 |
| ***Nitrogen*** | Total Nitrogen by wet oxidation (ppm) | 250m | Hengl et al., 2017 |
| ***Distance to Water*** | Distance to nearest available dry season surface water | 100m | this study – euclidean distance to the nearest waterhole (rivers treated as series of waterholes spaced 100m apart) |
| ***Water Density*** | Relative density of surface water per 100m^2^ |  | this study - kernel density estimation algorithm with 10.000m radius and a quartic (biweight) kernel |
| **ANTHROPOGENIC** |  |  |  |
| ***Distance to Settlement*** | Distance to nearest human settlement as a proxy of anthropogenic impact | 100m | this study – euclidean distance to the nearest point vector of house |
| ***Settlement Density*** | Relative density of houses per 100m^2^ | 100m | this study - kernel density estimation algorithm with 10.000m radius and a quartic (biweight) kernel |

**Telemetry data post-processing**

In our post-processing of the telemetry data, we filtered for impossible movements (such as spikes in speed >50 km/h) and retained only fixes with a low Positional Dilution of Position (PDOP<10) (Frair, Fieberg et al. 2010). We randomly selected one fix per day per collared lion to obtain independent detection and to standardize across collars featuring different position recording rate of collars. This temporal resolution of locations is considered sufficient for examining large scale processes such as residency or transience (Weston, Whitfield et al. 2013).

**Selecting resident data**

Telemetry data extended in some cases over multiple years, during which the tracked individuals could progress through demographic stages and disperse. To exclude non-residential movement, we used a biologically informed threshold based on the expected movement distances and maximum excursion duration of 10 breeding and therefore resident females in the study area following (Finerty, Cushman et al. 2023). We identified focal home range centres which we measured as the centroids of the 95% minimum convex polygons. Using the package “adehabitatLT” (Calenge 2011) in R we calculated Net Squared Displacement (Bunnefeld, Börger et al. 2011) for each individual from their starting location. Data was labelled as dispersal once the Net Distance of an individual exceeded the maximum ranging distance and the individual did not return within usual ranging distance for a period of one month (see Fig. S1, Supporting Information for example).


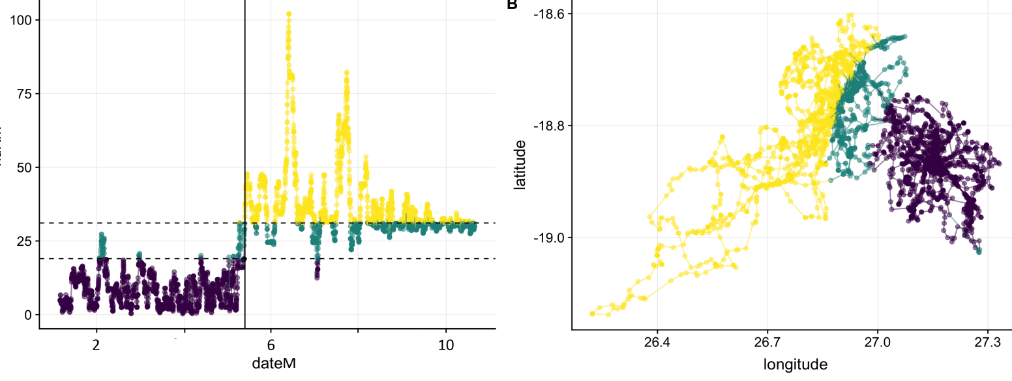


**Figure S1** Net Distance of a sub-adult male pre- and post dispersal.

Example of natal dispersal of a sub-adult male. The two dashed lines represent usual and maximum ranging distance (bottom and top) from the centroid of the animals 95% MCP. The vertical line represents date of dispersal (in months since the beginning of the study). Purple dots showcase data < 98^th^ percentile values, green dots values between 98^th^ percentile and maximum, and yellow dots represent data > maximum ranging distance for residents (roughly corresponding to resident, exploratory and dispersal movement respectively)

| **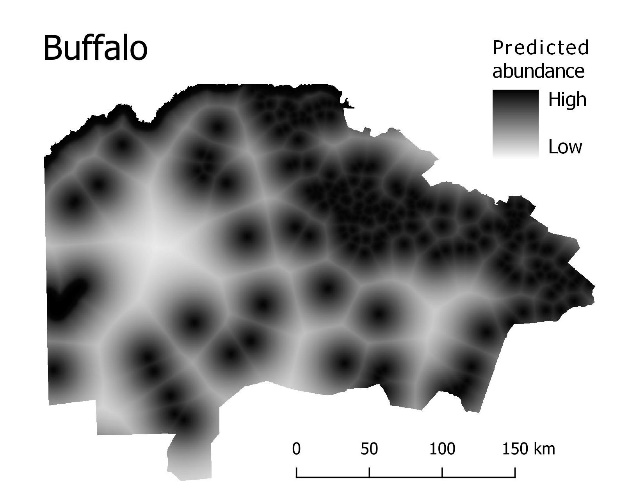** | **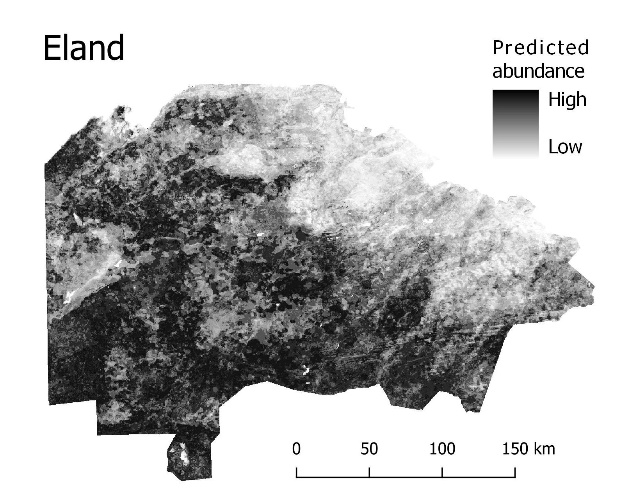** |
| --- | --- |
| **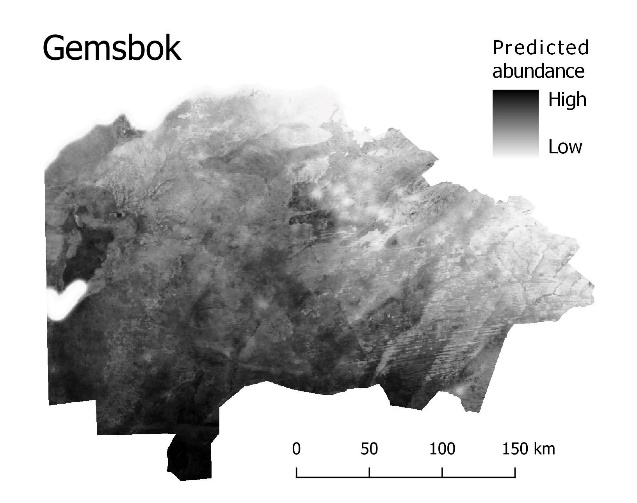** | **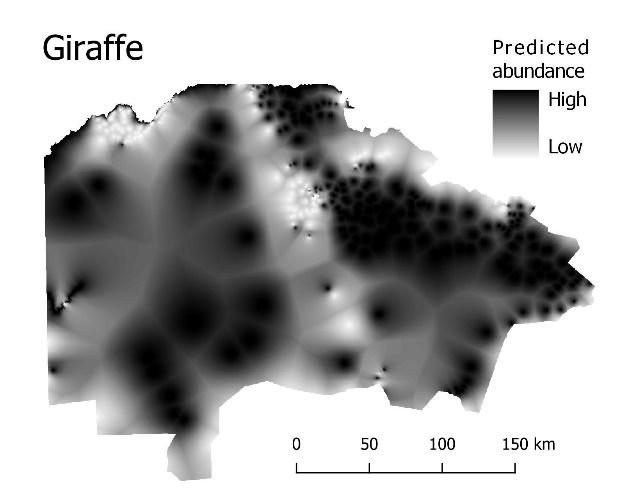** |
| **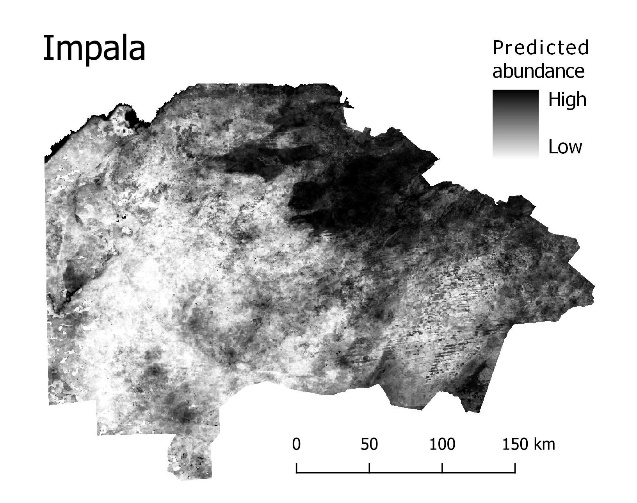** | **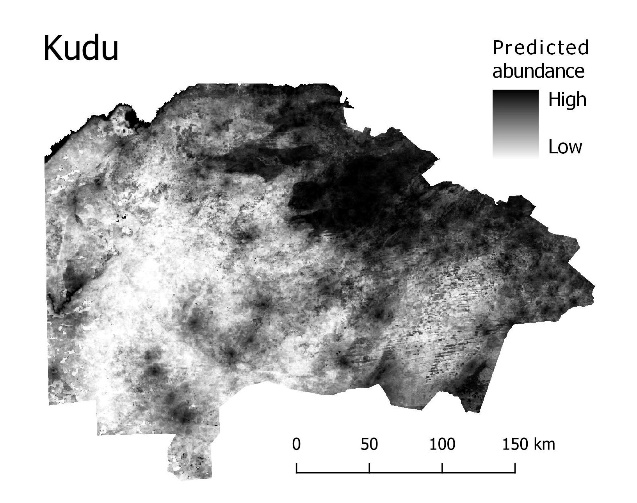** |
| **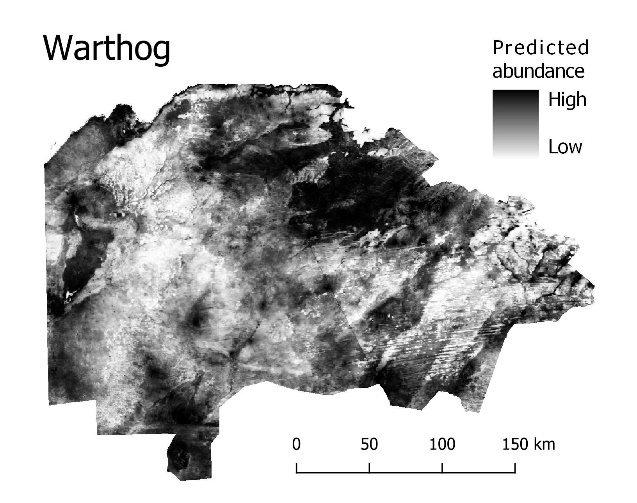** | **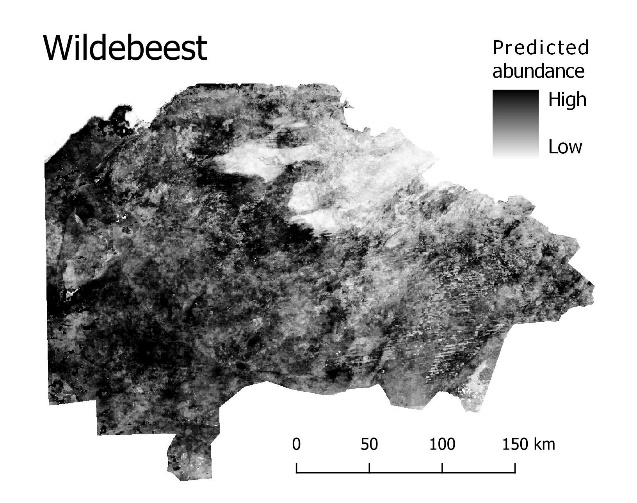** |
| **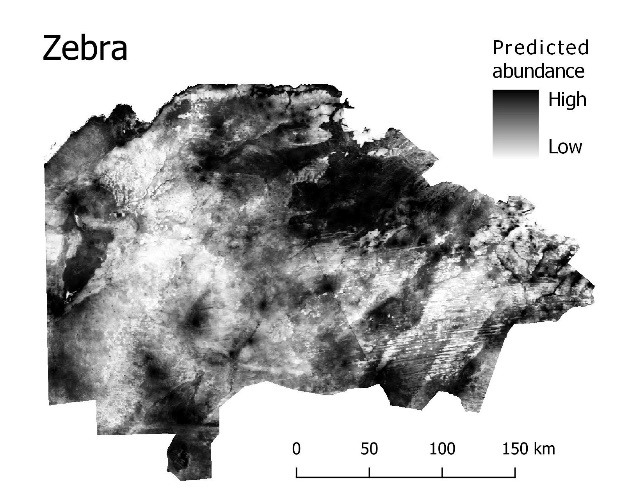** |  |

Figure S2 Predicted abundances for nine primary prey species of lions

**
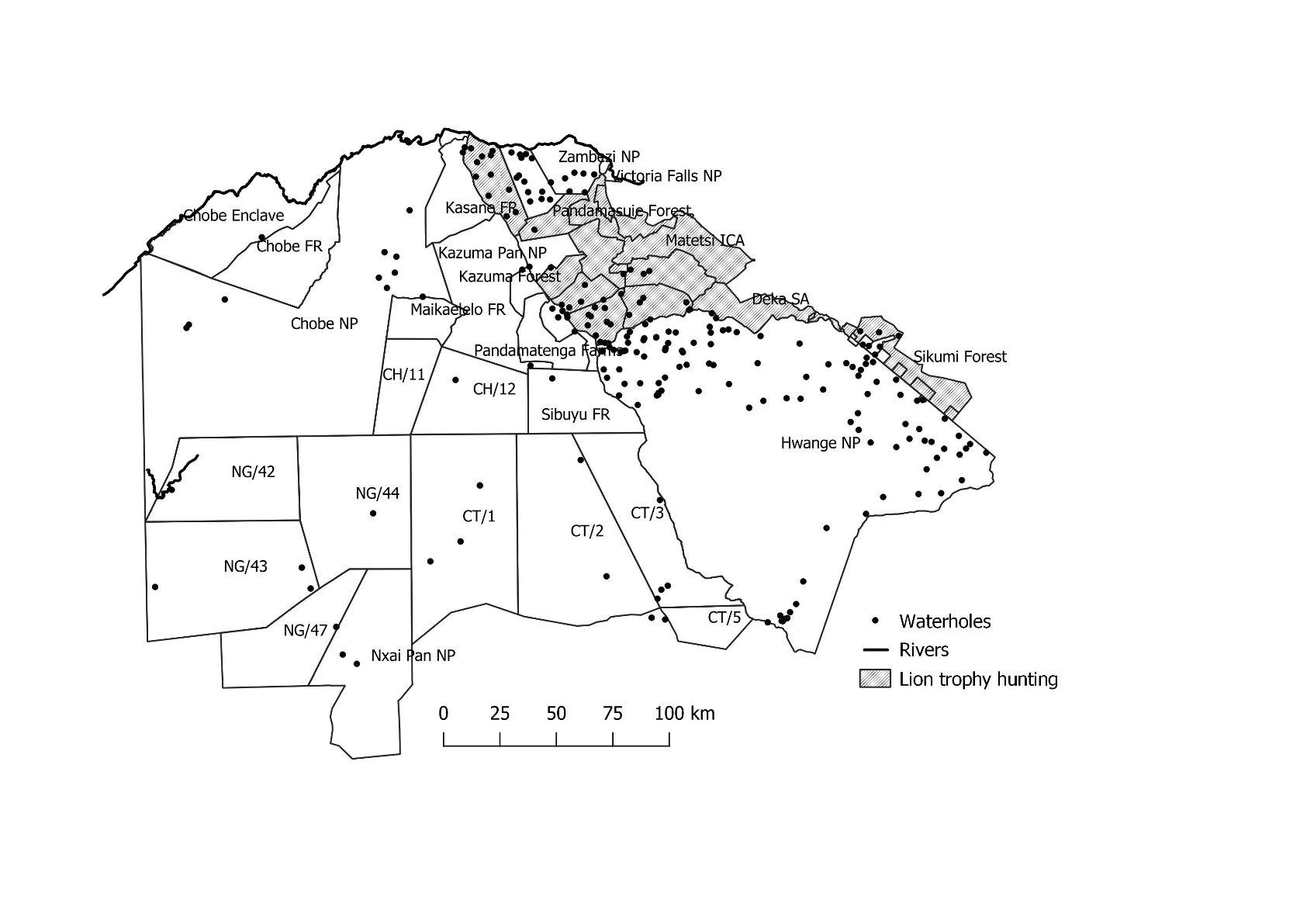
**

Figure S3 Artificial waterholes, permanent rivers and lion hunting areas

**References**

Bunnefeld, N., et al. (2011). "A model‐driven approach to quantify migration patterns: individual, regional and yearly differences." Journal of Animal Ecology **80**(2): 466-476.

Calenge, C. (2011). "Analysis of animal movements in R: the adehabitatLT Package." R Foundation for Statistical Computing: Vienna, Austria.

Finerty, G. E., et al. (2023). "Evaluating connectivity models for conservation: insights from African lion dispersal patterns." Landscape Ecology **38**(12): 3205-3219.

Frair, J. L., et al. (2010). "Resolving issues of imprecise and habitat-biased locations in ecological analyses using GPS telemetry data." Philosophical Transactions of the Royal Society B: Biological Sciences **365**(1550): 2187-2200.

Weston, E. D., et al. (2013). "When do young birds disperse? Tests from studies of golden eagles in Scotland." BMC Ecology **13**(1): 42.
